# Editorial: ILC checkpoints: expression and functions in physiological and pathological conditions

**DOI:** 10.3389/fimmu.2023.1220350

**Published:** 2023-05-22

**Authors:** Francesca Romana Mariotti, Nadia Guerra, Ana Stojanovic, Linda Quatrini

**Affiliations:** ^1^ Tumor Immunology Unit, Bambino Gesù Children’s Hospital, IRCCS, Rome, Italy; ^2^ Department of Life Sciences, Imperial College London, London, United Kingdom; ^3^ Department of Immunobiochemistry, Medical Faculty Mannheim, University of Heidelberg, Mannheim, Germany; ^4^ Mannheim Institute for Innate Immunoscience (MI3), Medical Faculty Mannheim, University of Heidelberg, Mannheim, Germany; ^5^ Innate Lymphoid Cells Unit, Bambino Gesù Children’s Hospital, IRCCS, Rome, Italy

**Keywords:** innate lymphoid cells, natural killer cells, activating receptors, inhibitory checkpoints, cancer

The family of Innate Lymphoid Cells (ILCs) is a heterogeneous group of cells that represent the innate counter part of the adaptive T lymphocytes and play essential roles against tumors and viral infections. ILCs comprise five different subsets: Natural Killer (NK) cells, helper innate lymphoid cells (ILC1, ILC2 and ILC3) and lymphoid tissue inducer cells (LTis), which are classified according to transcriptional factors expression, development, effector functions and cytokine production. NK cells and Group 1 ILC have common features, including the expression of the transcription factor T-bet and the production of Interferon-γ (IFN-γ) (1). While the anti-tumor effector function exerted by NK cells has been widely demonstrated, the roles played by ILC1 in this context are still being defined. ILC2, which are mainly involved in anti-helminth immunity, allergic inflammation and tissue repair, express the transcription factors Gata3 and RORα, and can secrete type 2 cytokines (2, 3). RORγt is instead required for the development of ILC3 subsets, that, according to the expression of natural cytotoxicity receptors (NCR), such as NKp46 and NKp44, can be further subdivided (4). ILC3s, which produce IL-22 and IL-17, as well as GM-CSF and IFN-γ, are mainly involved in tissue homeostasis against extracellular pathogens, while their role in the context of cancer is an emerging field of investigation. LTi cells are involved in the development of lymphoid organs during embryogenesis. Besides sensing cytokine micromilieus and tissue alarmins, ILCs rely on the expression of inhibitory and activating receptors to regulate their functions. In this regard, Khlifi et al. highlighted the role played by the activating receptors NCRs, KIR and CD16 in human NK cells in cancers. These receptors signal through association, as homo- or heterodimers, with adaptors bearing the immunoreceptor tyrosine-based activation motifs (ITAM), thus promoting NK cell effector functions. Increased expression of the NKp30a and NKp30b transcripts has been correlated with better prognosis and survival in paediatric neuroblastoma, in gastrointestinal tumors and hepatocellular carcinoma. In their review, authors further discuss the importance to boost tumor infiltration and effector function of NK cells through triggering of activating receptors with tumor-targeting monoclonal antibodies (mAbs). The recently developed antibody-based NK cell engager therapeutics (ANKET) platform therapy, consisting of specific NK cell engagers targeting NKp46 and CD16 with an antigen tumor tag in association or not with IL-2, has been shown to better promote NK cell activation and proliferation against CD20^+^ tumor cells, and in pediatric B-cell acute lymphoblastic leukemia.

It is well established that the tumor microenvironment (TME) and its cytokine milieu can differentially affect anti-tumor effector cells. In this context, the work by Siegler et al. demonstrates that in a context unfavourable for NK cell activity, ILC3s can exert cytotoxic effector function. Indeed, in the presence of a type 3 microenvironment, freshly-isolated circulating ILC3s, known to display multipotent phenotype, were able to stably express RORγt and produce IL-22, GM-CSF and IL-8. Further increase in IFN-γ release was observed in the presence of hepatoblastoma and hepatocellular carcinoma cell lines. Importantly, ILC3s killing of tumor targets was dependent on a cell-cell contact, and occurred through the TRAIL-TRAILR2 pathway, leading to Caspase-8 activation. Therefore, considering also the residency of ILC3s, this cell subset could provide a first-line of defence in early tumor development, even before NK cell recruitment, and contribute to anti-tumor immunity in TME that can be hostile for NK cell function.

ILC2s are the innate counterpart of CD4^+^ Th2 cells, and are involved in the maintenance of homeostasis in many tissues, including lung, skin, adipose tissue and small intestine. Whether their role in the context of cancer is protective or not, is not clear yet. Many contradictory results have been reported to date, suggesting that ILC2s contribution to the anti-tumor immune response is dependent on the tumor type and microenvironment. Besides their role in cancer pathogenesis, ILC2s may also contribute to the response to checkpoint inhibitors, because they express many checkpoint molecules upon activation. In particular, it was reported that the alarmin IL-33 activates ILC2s and induces the expression of the checkpoint PD-1 (5, 6). Howard et al. demonstrate that ILC2s in the lung up-regulate PD-1 expression in a B16 melanoma mouse model. In this tumor model, they showed that ILC2s were protective, and their adoptive transfer into Rag/γc-deficient mice reduced tumor burden, with an even higher efficiency when PD-1^-/-^ ILC2s were used. The authors show that IL-33-activated ILC2s release TNFα through an NFkB-dependent pathway, and TNFα had a cytotoxic effect on B16 melanoma cells. They demonstrate that PD-1 inhibits TNFα production, and tumor progression is inhibited *in vivo* by blocking this checkpoint. Moreover, the authors confirmed that PD-1/TNFα inhibitory pathway was also functional in human ILC2s activated *in vitro* with IL-33.

In the present issue, Painter and Akbari also revised the role of ILC2s in another pathological context, type 2 Diabetes mellitus (T2DM). This disease is characterized by the inability to maintain healthy glucose levels due to systemic insulin resistance. ILC2s have a protective role in this context, because they contribute to the maintenance of homeostasis in adipose tissue, which is a determinant in the metabolic outcome. Among the negative regulators of adipose ILC2s, the authors mention again PD-1. In obesity, this checkpoint is upregulated on ILC2s in response to TNFα, which, in turn, recruits inflammatory macrophages expressing PD-L1, thus further contributing to dampen ILC2s anti-inflammatory function (7). Another negative regulator of adipose ILC2s activation is adiponectin, which signals *via* AMPK (8). The authors also mention positive regulators of ILC2s response: GITR and DR3. GITR KO mice transferred with ILC2s and treated with a monoclonal agonistic antibody targeting GITR, showed improvements in glucose tolerance, suggesting that activation of adipose ILC2s through GITR may lead to an anti-inflammatory state able to prevent metabolic diseases, such as T2DM. Similarly to GITR, engagement of another TNF family receptor, DR3, ameliorated glucose intolerance, reversed insulin resistance and increased ILC2s effector functions, including IL-5 and IL-13 production. The authors propose that GITR and DR3 may be promising targets in T2DM, because antagonizing these receptors makes the adipose microenvironment shift towards a more Th2 cytokine profile.

For NK cells, our deep understanding of the expression and signalling of activating and inhibitory receptors in precise contexts, such as specific cancer types, has recently allowed to develop novel therapeutic strategies to promote NK cell response. For helper ILCs, we are just beginning to understand the role of checkpoints that function as “sensors” of the microenvironment, and can diminish or increase ILC functions locally. The collection of articles presented in this Research Topic highlights the diversity of immune checkpoints expressed by NK cells and ILCs, and novel functions and roles for immune checkpoints on ILC3s and ILC2s, thus contributing to our knowledge in this emerging field.

## Author contributions

All authors listed, have made substantial, direct and intellectual contribution to the work, and approved it for publication.
